# A survey exploring caregiver burden and health-related quality of life in hereditary transthyretin amyloidosis

**DOI:** 10.1186/s13023-022-02601-5

**Published:** 2023-01-26

**Authors:** Sarah Acaster, Siu Hing Lo, Sandra Nestler-Parr

**Affiliations:** 1 Acaster Lloyd Consulting Ltd, 8th Floor, Lacon House, 84 Theobalds Road, London, WC1X 8NL UK; 2 Akcea Therapeutics, 22 Boston Wharf Road, Boston, MA 02210 USA

**Keywords:** Transthyretin amyloidosis, Caregiver burden, Health-related quality of life, Utility values, Disutility

## Abstract

**Background:**

Hereditary transthyretin amyloidosis (ATTRv) is an ultra-rare, life-shortening disease with a high unmet need. This study examined ATTRv caregiver health-related quality of life (HRQoL) and productivity.

**Methods:**

A cross-sectional online survey, including EQ-5D-3L, Hospital anxiety and depression scale (HADS), and caregiver and patient characteristics questions, was developed to assess ATTRv caregiver burden. A companion general population survey collected EQ-5D-3L, HADS and chronic health conditions data. Caregiver-control group differences in HRQoL were assessed using t-tests and chi-square tests. Ordinary Least Squares regression was used to estimate the disutility of being a caregiver compared to controls stratified by patient ambulatory status.

**Results:**

Thirty-six caregivers and matched controls completed the survey (n = 72). The disease severity of patients they cared for was varied: 33% required no assistance walking, 58% required assistance with walking and 9% required a wheelchair/were bedridden. On average, caregivers spent 6 h daily on practical care and 4 h daily on emotional support. Fifty-six percent indicated that they had changed their employment due to providing ATTRv care. Caregivers reported lower HRQoL, as indicated by lower EQ-5D 3L utility scores (M = 0.772, SD = 0.178 vs. M = 0.849, SD = 0.218) and higher HADS anxiety (9.3 vs. 6.1, p < 0.01) and depression (7.6 vs. 4.4, p < 0.01) scores, compared with matched controls. Caregivers were also more likely to report sleep problems (33% vs. 8%, p < 0.01) and stress (42% vs. 0%, p < 0.001) as chronic conditions than controls.

**Conclusions:**

The study results indicate that caring for a person with ATTRv can have a considerable negative impact on caregivers’ HRQoL and productivity. The study findings provide important information for economic evaluations of ATTRv treatments.

## Background

Hereditary transthyretin amyloidosis (ATTRv) is an ultra-rare genetic disorder characterised by deposits of misfolded transthyretin (TTR) protein in the body’s organs and tissues. Build-up of TTR protein deposits lead to a diverse clinical presentation of neurological and/or cardiac symptoms with varying degrees of progressive disease, typically resulting in premature death. Neurological symptoms include peripheral neuropathy (sensory abnormalities, motor weakness, loss of ambulation) and autonomic dysfunction (severe gastrointestinal [GI] symptoms, cardiac arrythmias, orthostatic hypotension, bladder dysfunction), with malnutrition and wasting causing death. Cardiac complications include cardiomyopathy (typically thickening of the ventricular walls) resulting in heart failure.

Recent qualitative and quantitative research has described the profound impact of ATTRv on patient’s health-related quality of life and the considerable caregiver burden of ATTRv [1–3]. Relatedly, previous patient and caregiver surveys have also demonstrated that the caregiver burden of ATTRv was also high [4]. One study found a high number of hours spent on care. Furthermore, caregiving was associated with a considerable impact on work productivity as well as poor physical and mental health. Policy makers and health technology appraisal (HTA) agencies, such as the National Institute for Health and Care Excellence (NICE) in the UK, have recognised the need to consider caregiver burden, where relevant, when reviewing cost effectiveness of new treatments [5]. To quantify treatment benefits for caregivers in cost-effectiveness analyses, preference-based measures, such as the EQ-5D, are required to calculate utility (or disutility) values associated with caregiving (e.g. Acaster et al. [6]; Landfelt et al. [7]). As an example of the growing recognition of the importance of caregiver burden, reviews of the use of caregiver utilities in NICE technology appraisals (TAs) show an increase over time from zero in 2005 [8], rising to two in 2008 [9], six in 2012 [10] and 16 as of January 2019 [11]. The most recent review of NICE appraisals also highlights the growing importance of the application of carer utilities as it was commissioned by NICE to review and critique the methods being applied, shortly before they announced their HTA evaluation methods review.

To date, no caregiver (dis)utility data have been reported for ATTRv. The caregiver burden has also not been isolated from that of being a patient, as a high proportion (40%) of caregivers in the Stewart et al. [4] study were also diagnosed with ATTRv themselves. To obtain robust caregiver utilities and broader caregiver burden data, the caregiver impact needs to be disentangled from that of being a patient. Estimating the burden associated with caregiving for an ATTRv patient would also benefit from a matched population norm comparator to account for demographic variations in health unrelated to caregiving. Finally, the impact of patient disease severity on caregiver burden remains unexplored in ATTRv. Pennington and Wong’s [11] review of NICE appraisals emphasised the need to explore caregiver health-related quality of life (HRQoL) changes over time, including when the patient’s health improves or worsens, to increase accuracy of health economic models. Thus, understanding how disease severity impacts caregiver burden is particularly relevant in the context of a progressive disease such as ATTRv, with patients able to walk unassisted at disease onset but needing a wheelchair or being bedridden in the final stages of disease.

The present study aimed to extend and refine the caregiver impacts reported by Stewart et al. (2018) to provide a more robust and detailed understanding of the ATTRv caregiver burden, including caregiver utility data suitable for inclusion in economic analyses. More specifically, this study aimed to capture caregiver burden using validated measures of health-related quality of life (EQ-5D), anxiety and depression (Hospital anxiety and depression scale) and bespoke assessments of time spent providing care, career impacts and chronic health conditions. Caregiver health impacts were estimated by including a matched general population sample and isolating caregiver impact from any patient impact by accounting for the ATTRv status of caregivers. Finally, the impact of ATTRv disease severity, as classified by Coutinho stages of ambulatory status [12], on caregiver burden was also explored.

## Methods

### Study design

A cross-sectional online survey was developed to assess the caregiver burden of ATTRv. The survey was reviewed by Amyloidosis Research Consortium (ARC) UK and piloted using feedback from one caregiver in the ARC UK network. Pilot data were included in the analysis. A companion general population survey was designed to collect data to compare with the caregiver data where relevant.

### Sample and participant recruitment

Thirty-six ATTRv English-speaking caregivers were recruited via ARC UK and a healthcare panel agency in the UK, US, Canada, Australia and New Zealand. Caregiver inclusion criteria were: (i) currently providing regular (at least weekly) informal (unpaid) care for someone with ATTRv; (ii) residing in the UK, US, Canada, Australia, or New Zealand; and (iii) being aged eighteen or over. Data was collected across a number of English-speaking countries to achieve the largest possible sample across populations that are comparable. Country differences in the caregiver data were not examined, as the small sample size precluded meaningful group comparisons between countries.

A matched general population sample (n = 36) was recruited in the UK via the same healthcare panel agency. The general population sample inclusion criteria were: (i) not providing informal (unpaid) care for someone with a disease or disorder on a regular (at least weekly) basis; (ii) residing in the UK; and iii) being aged eighteen or over. The general population sample was matched with the caregiver sample on the group-level in terms of gender, age group (18–24/25–34/35–44/45–54/55–64/65 or over), living situation (living with partner or spouse / other living arrangements) employment status (employed full-time/employed part-time/unable to work due to own health/unemployed or homemaker or retired or other).

### Measures

The survey included items related to caregiver burden, caregiver proxy-reported patient characteristics, socio-demographics and chronic health conditions. The survey also included two validated questionnaires: the EQ-5D-3L and the Hospital anxiety and depression scale (HADS). Study-specific measures and the validated questionnaires are described below.

#### Caregiver burden and characteristics

The caregiver sample completed caregiver burden measures including (i) hours of informal care, broken down by time spent on practical care and emotional support; and (ii) employment impact, defined as change in employment status due to ATTRv care (stopped working/reduced hours/changed jobs/other, please specify).

Caregivers were also asked about (iii) the number of ATTRv patients they were caring for; (iv) the relationship between the caregiver and the ATTRv patient(s); and (vi) how long they had been a caregiver for.

#### Patient characteristics

Caregivers were asked to proxy-report patient characteristics including (i) time since diagnosis; (ii) type of ATTRv (cardiomyopathy/polyneuropathy/both/other or not sure); (iii) V-30 M status (yes/no/not sure); (iv) ambulatory status using Coutinho staging (no assistance with walking/assistance walking/wheelchair/bedridden); (v) symptoms, including a list of 7 neuropathic, 2 cardiac, 4 gastrointestinal and 8 other symptoms; (vi) patient demographics.

#### Socio-demographic and chronic conditions

All (i.e. caregiver, caregiver-patient and general population) participants completed background questions on (i) socio-demographics; and (ii) chronic health conditions, which included a list of common chronic conditions (arthritis, anxiety, cancer, chronic kidney disease, depression, diabetes, digestive pain/discomfort, heart disease, hypertension [high blood pressure], respiratory condition [e.g. asthma, COPD], sleep problems, stress) and an open-ended ‘other’ response option.

#### EQ-5D-3L

All (i.e. caregiver, caregiver-patient and general population) participants completed the EQ-5D-3L questionnaire. The EQ-5D-3L is a generic health status measure [13]. Participants indicate their current health status on five domains (mobility; self-care; usual activity; pain/discomfort; anxiety/depression) as either no problems, some problems or severe/extreme problems. Participants also indicate their current health on a visual analogue scale. Health utilities were derived from the EQ-5D using UK general population preference weights[14], which provide a potential range of scores from − 0.59 to 1.0 (where a score of 1 represents full health and a score of 0 represents dead).

#### HADS

All (i.e. caregiver, caregiver-patient and general population) participants completed the HADS questionnaire. The HADS is a valid and reliable self-rating scale that measures anxiety and depression in both hospital and community settings [15]. The questionnaire gives clinically meaningful results as a psychological screening tool and can assess the symptom severity and “caseness” of anxiety disorders and depression in patients with illness and the general population. The HADS consists of fourteen items; seven questions for anxiety and seven for depression. The responses being scored on a scale of 0–3 (3 indicating higher symptom frequencies). Scores for each subscale (anxiety and depression) range from 0 to 21 with scores categorised as normal (0–7), mild (8–10), moderate (11–14) and severe (15–21). Moderate and severe scores are equated with ‘probable clinical caseness’; UK population norms have been reported for such levels of anxiety and depression [16].

### Statistical analysis

General population and caregiver socio-demographics, caregiver burden, caregiver characteristics, and patient characteristics were tabulated using descriptive statistics.

Caregiver burden and characteristics were also analysed by patient ambulatory status (Coutinho stages, with ‘requires wheelchair’ and ‘bedridden’ collapsed due to small cell sizes) using independent t-tests and chi-square tests as appropriate. For caregivers who cared for multiple ATTRv patients (n = 2), the worst (more severe), or if equal severity the first, reported patient’s ambulatory status was used in all analyses.

Caregiver HADS anxiety and depression scores were compared with matched general population scores and by patient ambulatory status using independent t-tests. Group differences in the proportion of moderate/severe anxiety and depression (‘probable clinical cases’; score range:11–21) were tested for using chi-square tests.

Frequencies and distributions of the EQ-5D profile scores were described using bar charts for the general population and caregiver samples. Utility scores were calculated from the EQ-5D 3L data based on the UK valuation weights [14]. Ordinary least squares regression was used to estimate the disutility associated with being a caregiver, stratified by Coutinho stage, versus the matched general population sample. Due to small sample sizes the ‘requires wheelchair’ and ‘bedridden’ stages were collapsed, giving four groups in total: matched control (general population), requires no assistance walking, requires assistance walking, and requires wheelchair/bedridden, with controls as the reference category. The model was run with and without socio-demographic variables as covariates, as the caregivers and controls were only matched at the overall group level.

The caregiver sample included only one patient-caregiver (n = 1), i.e. a caregiver who was also an ATTRv patient, who was the only participant unable to work due to ill health in the caregiver sample. All descriptive statistics are provided for the overall caregiver sample, with patient-caregiver responses described in table footnotes where relevant. All statistical tests were run with and without the patient-caregiver and one general population matched control respondent (n = 1) who, like the patient-caregiver, was unable to work due to ill health. Only test results using the overall sample were reported, unless results with and without patient-caregiver and matched control differed.

All statistical tests were two-sided and p-values below 0.05 were considered statistically significant.

## Results

### Sample characteristics

A total of 36 English-speaking caregivers and 36 matched controls completed the online survey. Of the total caregiver sample, 6 were recruited via ARC-UK and 30 via a healthcare panel agency. One participant dropped out after completing the informed consent form, resulting in a dropout rate of 3%. No general population participants dropped out. Of the 36 caregivers, 23 (64%) resided in the United States, 8 (22%) in the United Kingdom, 3 (8%) in Canada, 1 (3%) in Australia and 1 (3%) in New Zealand.

Over three-quarters (78%) of caregivers in the sample were caring for a partner or spouse, with the remainder caring for a sibling (14%), parent (3%) or child (3%). Only one (3%) caregiver had indicated she had ATTRv herself.

Caregiver and general population sample characteristics are described in Table 1. Most caregivers were female (81%) and had a mean age of 56.3 years (SD = 12.5). The vast majority of caregivers were living with a partner or spouse (94%). The caregiver sample was varied in terms of employment status, with 33% retired, 28% part-time employed and only 14% full-time employed. As expected due to the sampling methodology, the caregiver and general population sample characteristics were comparable in terms of age, gender, living situation and employment status.

**Table 1:** Caregiver sample characteristics and proxy-reported patient characteristics

Sample characteristic	Caregivers^a^ (N = 36)	General population^b^ (N = 36)
	Mean (SD)	Mean (SD)
Age	56.3 (12.5) ^a^	57.5 (13.4) ^b^

	N (%)	N (%)
Gender: female	29 (81%) ^a^	29 (81%) ^b^
Living situation: with partner/spouse	34 (94%)	36 (100%) ^b^
Living situation: with relative	2 (5%) ^a^	0 (0%)
Employment status: employed full-time	5 (14%)	5 (14%)
Employment status: employed part-time	10 (28%)	10 (28%)
Employment status: homemaker/retired/unemployed/other	20 (56%)	20 (56%)
Employment status: unable to work due to own health	1 (3%) ^a^	1 (3%) ^b^

Patient characteristic	Mean (SD)	
Time since diagnosis (years)	5.76 (4.80) ^a^	
Age	59.00 (13.38) ^a^	

	**N (%)**	
Gender: male	28 (78%) ^a^	
Employment status: employed, full- or part-time	6 (17%)	
Ambulatory status*: requires no assistance walking	12 (33%)	
Ambulatory status*: requires assistance with walking	21 (58%)	
Ambulatory status*: requires wheelchair	2 (6%)	
Ambulatory status*: bedridden	1 (3%) ^a^	
Type of ATTRv: cardiomyopathy	2 (6%)	
Type of ATTRv: polyneuropathy	11 (31%)	
Type of ATTRv: cardiomyopathy and polyneuropathy	20 (56%) ^a^	
Type of ATTRv: other/not sure	3 (8%)	
V-30 M status: V-30 M variant confirmed	8 (22%)	
Symptoms: neuropathic symptom(s) reported	36 (100%) ^a^	
Symptoms: cardiac symptom(s) reported	26 (72%) ^a^	
Symptoms: gastrointestinal symptom(s) reported	30 (83%) ^a^	

^a^Characteristics of the patient-caregiver aged 38 (n=1) and the patient aged 46 who the patient-caregiver was caring for

^b^Characteristics of the matched control aged 38 (n=1)

*Requires no assistance walking, requires assistance with walking and requires wheelchair correspond to Coutinho stages 1, 2 and 3 respectively

Compared with the general population sample, caregivers were more likely to report sleep problems (caregivers: 33% vs. gen. pop.: 8%, $\chi$ ^2^ = 6.8, p < 0.01) and stress (caregivers: 42% vs. gen. pop.: 0%, $\chi$^2^ = 19.0, p < 0.001) as chronic health conditions. There were no statistically significant differences between caregiver and general population samples in the proportion who reported anxiety, depression and common physical health diseases as chronic health conditions.

### Patient characteristics

Patient characteristics were proxy-reported by the caregiver sample and are shown in Table 1. The ATTRv patients the caregiver sample cared for were on average diagnosed 5.8 years (SD = 4.8) ago. Most were male (78%) and had a mean age of 59.0 (SD = 13.4). Only 17% of patients were part-time or full-time employed. One-third (33%) of patients could walk unassisted, over half (58%) required assistance walking, 6% required a wheelchair and 3% were bedridden. Over half (56%) reported that the patient had cardiomyopathy and polyneuropathy, around one-third (31%) had reported polyneuropathy and 6% had reported cardiomyopathy. Approximately one-fifth (22%) reported a V-30 M variant. When asked to report specific symptoms the patients experienced, all (100%) reported one or more neuropathic symptom(s) and most also reported gastrointestinal symptom(s) (83%) and cardiac symptom(s) (72%).

### Caregiver burden

#### Informal care and impact on caregiver employment

Overall, caregivers spent an average of 5.8 h on practical care (median = 3.0; SD = 6.2) and 4.1 h on emotional support (median = 2.0; SD = 6.1) on a daily basis (Table 2). Hours spent on practical care was significantly associated with patient ambulatory status, with those with more severe impairment requiring more practical support (p < 0.05). In contrast, hours spent on emotional support was not associated with patient ambulatory status.

**Table 2:** Hours of practical and emotional care per day

***Practical care***^***a***^								
Total sample	N	Mean	Median	SD				
	36	5.79^a^	3.00	6.22				

By ambulatory status	N	Mean	Median	SE	Lower bound 95% CI	Upper bound 95% CI	t	p
No assistance walking	12	2.64	2.00	0.54	1.45	3.83	Ref	
Assistance with walking	21	6.88	4.00	1.41	3.95	9.82	− 2.21	0.03
Wheelchair/bedridden	3	10.67^a^	5.00	6.69	− 18.13	39.46	− 2.56	0.02

***Emotional support***^***a***^								
Total sample	N	Mean	Median	SD				
	35*	4.08	2.00	6.13				

By ambulatory status	N	Mean	Median	SE	Lower bound 95% CI	Upper bound 95% CI	t	p
No assistance walking	12	3.56	1.07	1.49	0.28	6.84	Ref	
Assistance with walking	20	4.74	2.00	1.58	1.43	8.05	− 0.50	0.62
Wheelchair/bedridden	3	1.76^a^	1.00	1.14	− 3.13	6.66	0.57	0.57

^a^The patient-caregiver (n=1) reported providing 24 hours of practical care and 4 hours of emotional support per day

*Sample size is 1 less than elsewhere because the emotional support item was added after the pilot

**Fig. 1:**
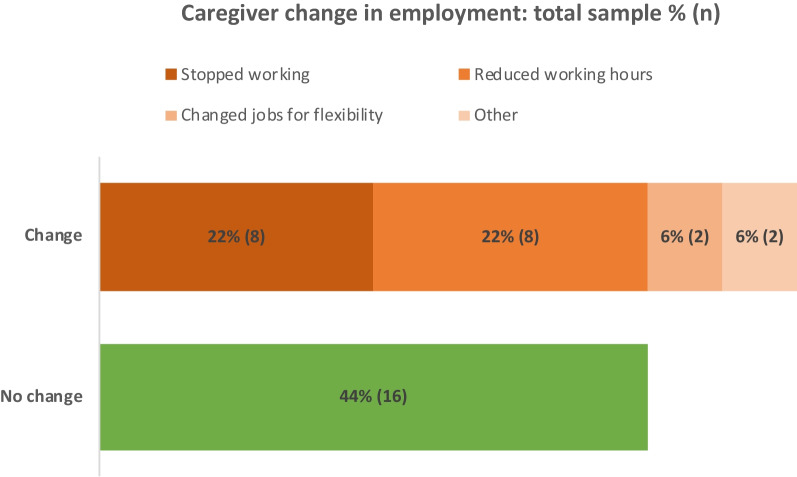
Caregiver change in employment as a result of caregiving for ATTRv patient

The majority of caregivers were not employed, with only 42% in either full-time or part-time employment. Over half (56%) of caregivers had indicated that they had changed their employment as a result of ATTRv care; Fig. 1 shows a breakdown of caregiver change in employment. Caregiver change in employment was significantly associated with patient ambulatory status, with a higher proportion of caregivers who supported patients requiring assistance with walking reporting a change in employment compared with caregivers of patients who did not require assistance walking (71% vs. 42%, χ^2^ = 6.83, p = 0.03).

#### Impact on caregiver anxiety and depression

**Fig. 2:**
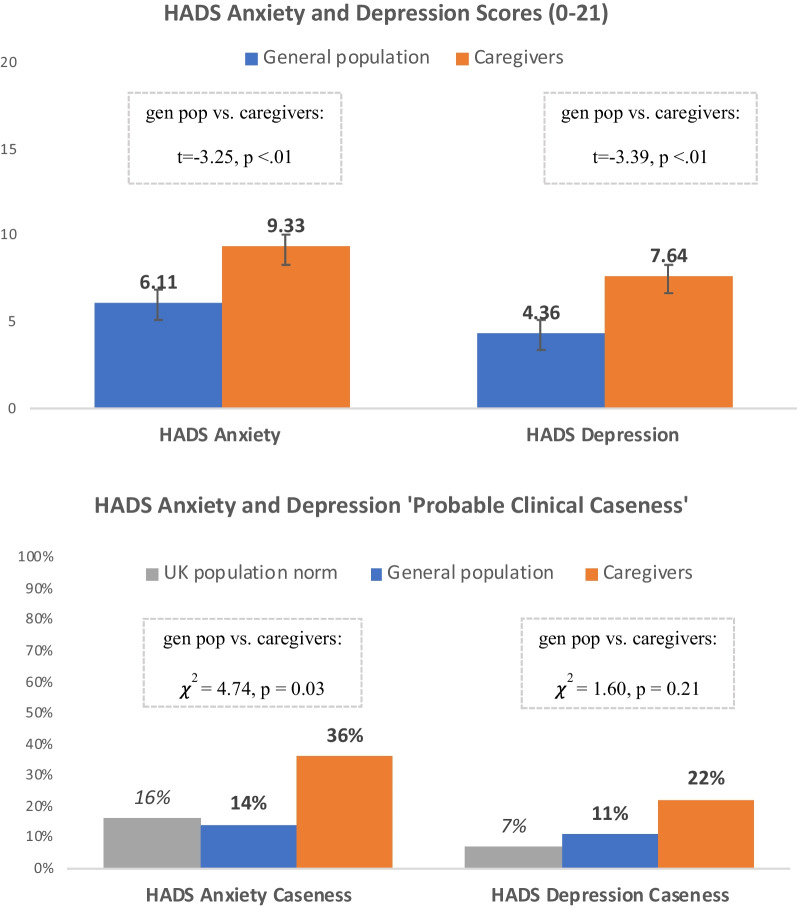
HADS Anxiety and Depression: caregivers vs. general population

As illustrated in Fig. 2, caregivers had significantly higher HADS anxiety scores than the matched general population (M = 9.3 vs. M = 6.1, t = -3.25, p < 0.01). Furthermore, a significantly higher proportion of caregivers had HADS anxiety scores qualifying as ‘probable clinical anxiety’ compared with the matched general population, which was comparable to the UK population norm data (36% vs. 14%, χ^2^ = 4.74, p = 0.03). Within the caregiver sample, patient ambulatory status was not significantly associated with caregiver HADS anxiety scores or the proportion with ‘probable clinical anxiety’.

Caregivers also had significantly higher depression scores than the matched general population (M = 7.6 vs. M = 4.4, t = − 3.39, p < 0.01; Fig. 2). While the proportion of caregivers with HADS depression scores qualifying as ‘probable clinical depression’ was higher than that in the general population matched controls, these group differences were not significantly different (22% vs. 11%, χ^2^ = 1.6, p = 0.21). Among caregivers, patient ambulatory status was not significantly associated with caregiver HADS depression scores or the proportion with ‘probable clinical depression’.

#### Caregiver health-related quality of life

**Fig. 3:**
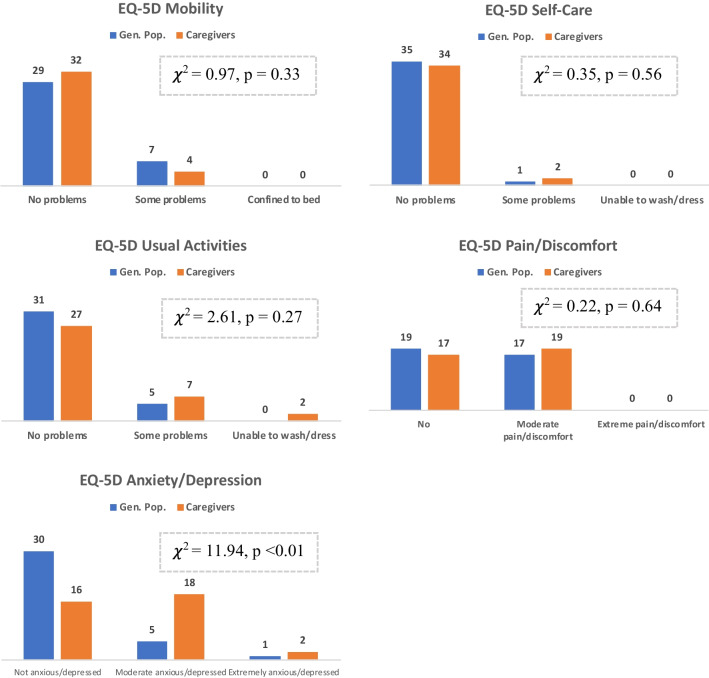
EQ-5D-3L Profiles: caregivers vs. general population. ^a^Profile of patient-caregiver: mobility = no problems; self-care = some problems; usual activities = some problems; pain/discomfort = moderate; anxiety/depression = moderate. ^b^Profile of matched control who was unable to work due to ill health: mobility = some problems; self-care = some problems; usual activities = some problems; pain/discomfort = moderate; anxiety/depression = extreme

Overall, caregivers reported lower HRQoL than socio-demographically matched members of the general population as measured using the EQ-5D-3L health utility values. Utilities values for the caregiver and general population groups and by patient ambulatory status for caregivers are shown in Table 3. All utility and estimated disutility values are given with and without data from the patient-caregiver and corresponding matched control. For the overall sample, the average caregiver utility score was 0.772 (SD = 0.178) compared with a matched general population utility of 0.849 (SD = 0.218). Excluding the patient-caregiver and corresponding matched control, average caregiver utility was 0.778 (SD = 0.219) compared with a general population utility of 0.871 (SD = 0.122). The caregiver-general population difference in utilities was statistically significant when excluding the patient-caregiver (b = − 0.094, 95% CI − 0.178–− 0.009, t = − 2.21, p = 0.03) but was not significant for the total sample. Further examination of the EQ-5D-3L profiles suggests that lower caregiver utility values were predominantly driven by lower caregiver scores on the EQ-5D-3L anxiety/depression domain ($\chi$^2^ = 11.9, p < 0.01; Fig. 3).

**Table 3:** EQ-5D-3L Utility and Disutility: general population, caregivers and caregivers by ambulatory status

***EQ-5D-3L utilities***				
Caregivers versus general population	N	Mean	SD	
Total sample				
General population	36	0.849	0.218	
Caregivers	36	0.772	0.178	

Excluding patient-caregiver^a^				
General population	35	0.871	0.122	
Caregivers	35	0.778	0.219	

Caregivers by ambulatory status	N	Mean	SD	
Requires no assistance walking	12	0.819	0.126	
Requires assistance with walking	21	0.754	0.266	
Requires wheelchair	2	0.787	0.087	
Bedridden^b^	1	0.585	–	

***Estimated EQ-5D-3L disutilities***^***C***^ ***by ambulatory status***				
Predictors in regression model	Without covariates		With covariates	
	Coefficient (disutility)	SE	Coefficient (disutility)	SE
Total sample				
General population	Ref		Ref	
Requires no assistance walking	− 0.031	0.067	− 0.036	0.066
Requires assistance with walking	− 0.096	0.055	− 0.101	0.055
Requires wheelchair/bedridden	− 0.130	0.121	− 0.110	0.121
Age	–	–	− 0.003	0.002
Gender	–	–	− 0.059	0.061
Not employed vs. employed (ref.)	–	–	− 0.053	0.053

Excluding patient-caregiver^a^				
General population	Ref		Ref	
Requires no assistance walking	− 0.053	0.060	− 0.036	0.066
Requires assistance with walking	− 0.118*	0.049	− 0.101	0.055
Requires wheelchair/bedridden	− 0.085	0.130	− 0.110	0.121
Age	–	–	− 0.003	0.002
Gender	–	–	− 0.059	0.061
Not employed vs. employed (ref.)	–	–	− 0.053	0.053

^a^Sample excluding patient-caregiver (n=1) and corresponding matched control (n=1).

^b^The caregiver utility value for the bedridden patient is that of the patient-caregiver (n=1).

^c^Estimated using Ordinary Least Squares (OLS) regression

*p<0.05

Estimated disutility values associated with providing care stratified by ambulatory status, using the general population as a reference category, are also provided in Table 3. As the general population were matched at the group level (rather than at the individual caregiver level), the regression model was repeated including socio-demographic covariates.

In line with the EQ-5D-3L utility values, caregivers also had lower EQ-5D VAS scores than the matched general population, although this did not reach statistical significance (caregivers = 75.7 vs. gen. pop. M = 81.4, t = 1.98, p = 0.051).

## Discussion and conclusions

The mental health impact of providing care was considerable. Anxiety levels as measured by the HADS showed that caregivers had significantly higher anxiety scores, reporting two and a half times higher levels of ‘probable clinical anxiety’ than matched controls. Notably, the levels of anxiety among controls in this sample were in line with previously published UK population norms for the HADS [16]. Caregivers also reported significantly higher HADS depression scores than controls. Caregivers were twice as likely to report ‘probable clinical depression’, although this was not statistically significant. In addition to higher levels of HADS anxiety and depression scores, caregivers were also significantly more likely to report sleep problems and stress as chronic health conditions than the matched general population sample.

These impacts were reflected in lower HRQoL, as indicated by lower EQ-5D-3L utility values among caregivers compared with matched controls, largely driven by anxiety / depression levels. Furthermore, the disutility associated with providing care increased (i.e. worse quality of life) as patient disease progressed, as measured by proxy-reported patient ambulatory status (Coutinho staging). These are the first ATTRv-specific caregiver disutility estimates suitable for supporting cost-effectiveness analyses of treatments for this rare, life-shortening disease. While the sample is small, these estimates are comparable to the caregiver burden reported in multiple sclerosis (MS), a neurological condition which is associated with progressive loss of physical functioning similar to ATTRv [6]. The published MS caregiver utility value (0.74) is comparable to the ATTRv caregiver utility (0.77) reported in this study. Caregiver utility values by disease severity also show a similar pattern in both conditions, with utilities decreasing as physical function declines up until the stage that the patient requires a wheelchair. Furthermore, both MS caregivers and ATTRv caregivers are more likely to report sleep problems and have HADS ‘probable clinical anxiety’ than matched controls. Given the small sample size in the present study, this provides added face validity to the utilities presented here.

Notably, despite the self-reported HADS and EQ-5D anxiety and depression ratings, caregivers were not more likely to report anxiety and depression as chronic health conditions than matched controls. This might be explained by a lack of a clinical diagnosis for those conditions, as their mental health symptoms can be attributed to caregiving for a loved one with a serious condition and as such do not require them to seek treatment. Future studies related to caregiver burden would benefit from exploring caregiver’s perception of the mental and physical impacts of providing care, and the extent to which they feel they need and/or seek help.

The results of this study are consistent with the ATTRv caregiver burden described in previous research, showing a considerable psychosocial caregiver burden and an association between the level of support the patient requires and caregiver burden [2–4]. Despite some differences in caregiver and patient characteristics between the two studies, Stewart et al. [4] reported the equivalent of an average of 6.6 h of informal care (all types of care) per day, which is roughly comparable to the average of 5.8 daily hours of practical care and 4.1 daily hours of emotional support reported in this study. However, it should be acknowledged that ATTRv caregiver burden could vary across regions due to different disease phenotypes [17]. A previous study conducted in Majorca, an endemic area of the disease, highlighted ATTRv autonomic symptoms as the main source of caregiver and family burden [2], whereas the present study highlighted the impact of loss of ambulation on caregiver burden.

The current study extended this previous research by estimating the caregiver impact with a preference-based measure, considering career impacts, using matched controls as a comparator where appropriate and accounting for the ATTRv status of caregivers. EQ-5D 3L utility data suited for supporting cost-effectiveness analysis and caregiver burden by disease stage were reported. The study further demonstrated higher rates of anxiety and depression, as indicated by HADS scores, in the ATTRv caregiver sample than in matched controls.

The results should nevertheless be interpreted in light of the study limitations. The sample sizes were small, thus decreasing the precision of estimates and limiting the statistical power to detect between-group differences. Relatedly, due to the extreme rarity of the disease in the UK, the caregiver sample was a convenience sample and no further inclusion criteria were set to ensure representativeness of the sample. Studies conducted in countries where ATTRv is endemic or where a national ATTRv registry exists may collect data from a larger sample [2, 3]. However, ATTRv is a heterogeneous disease and prevalence of ATTRv phenotypes differs between ATTRv-endemic and non-endemic countries, so some findings relating to the caregiver and patient burden of ATTRv may not be generalisable across countries [17]. The cross-sectional survey methodology also precluded any conclusions on the direction of causality, as any differences between disease stages may be influenced by between subject variance as well as disease stage.

To conclude, the present study aimed to examine ATTRv caregiver HRQoL and productivity in the UK. Compared with matched controls, caregivers reported lower HRQoL, and higher rates of anxiety, depression, stress and sleep problems. On average, caregivers reported spending 6 h daily on practical care and 4 h daily on emotional support. The study findings provide important information for economic evaluations of ATTRv treatments.

## Data Availability

Due to the nature of this research, participants of this study did not agree for their individual data to be shared publicly, therefore supporting data is not available.

## Abbreviations

ATTRv: Hereditary transthyretin amyloidosis
TTR: Transthyretin
GI: Gastrointestinal
HTA: Health technology appraisal
NICE: National Institute for Health and Care Excellence
TAs: Technology appraisals
HRQoL: Health-related quality of life
ARC: Amyloidosis Research Consortium
HADS: Hospital anxiety and depression scale
